# Contribution of Treatment with Ear Popper for Hearing in Children with Middle Ear Effusion

**DOI:** 10.3390/children11060744

**Published:** 2024-06-18

**Authors:** Ronit Priner, Ophir Ilan

**Affiliations:** 1 The Faculty of Communication Disorder, Hadassah Academic College, Jerusalem 9422408, Israel; ronitpr@edu.hac.ac.il; 2 Department of Otolaryngology—Head & Neck Surgery, Hadassah Medical Center, Jerusalem 91120, Israel

**Keywords:** EarPopper, middle ear effusion, tympanometry, auto inflation

## Abstract

Objectives: we aim to assess the contribution of the EarPopper device to hearing in children with middle ear effusion (MEE). Methods: The study has three parts, including 1. tympanometry and audiometry before and six weeks after using the EarPopper to evaluate the treatment’s effect over time compared to a control group; 2. tympanometry before and immediately after using the EarPopper to evaluate immediate changes in middle ear pressure (MEP); 3. length of effect 90 min after use to assess pressure fluctuations over time. Results: Part 1 was a follow-up six weeks after using the device, and the patients in the study group that completed the study showed a significant improvement in hearing threshold. The average gain in hearing threshold ranged from 9.1 dB to 14 dB compared to the control group’s max improvement of 1.1 dB. In addition, MEP was significantly improved in the study group, as most Type Bs improved to Type A and C. Part 2 was the tympanometry immediately after using EarPopper and showed the majority of Type Cs turned into Type As. The majority of Type Bs remained unchanged. Part 3 was a follow-up 90 min after use; Type Cs that had improved to Type A demonstrated a decrease in pressure and return to negative pressure. Conclusions: use of the EarPopper device for six weeks is associated with an improved hearing threshold and middle ear status.

## 1. Introduction

Dysfunction of the Eustachian tube (E.T.) has been found to have a causative role in middle ear (ME) diseases [1,2]. A common pathology caused by E.T. dysfunction is fluid retention in the ME space, termed middle ear effusion (MEE). This is the most common pathological finding diagnosed by pediatricians. It is believed to occur in up to 70% of the pediatric population (mainly in the first three years of life) [3,4]. MEE has a significant influence on the function of the ear. It dims the conduction of sound waves from the external environment to the inner ear, leading to mild to moderate conductive hearing loss, usually associated with Type B tympanometry [4,5]. Difficulties in language and speech development in children of three years of age or more are often the result of MEE and the associated hearing impairment. [6]. In addition, MEE causes difficulties in auditory processing [7]. Therefore, there is great importance in treating this impairment as early as possible.

Management of MEE is divided into non-surgical and surgical interventions, including 1. Valsalva, Politzer, or EarPopper maneuvers [5,8] and 2. ventilation tube surgery. Ventilation tube surgery improves the auditory thresholds considerably and rapidly [6] and helps in the prevention of MEE recurrence [9,10,11]. Complications are eardrum perforations; the longer the tube stays in place, the higher the risk of perforation [12,13]. Frequently, the tubes fall out and patients have recurrent MEE; 33–75% will undergo surgery again to replace the tubes [14]. The surgery itself and the fact it is performed under general anesthesia leads to parents’ anxiety (10% to 18% will cancel the surgery) [15].

The first method to manage E.T. dysfunction is inflating the E.T. and ME using the Valsalva or Politzer maneuver. The limitations of the Politzer maneuver include cumbersome design, failure to produce continuous and unfluctuating air flow and synchronize air pressure and swallowing, and lack of pressure and volume control of the air entering the nose. In addition, patients often failed to handle the pear properly, and the device was not convenient for home use. These flaws could be attributed to device design rather than to the fundamentals of the method [16]. The flaws of the Politzer maneuver led to the development of a device based on the same fundamentals—the EarPopper.

According to the manufacturer’s description, the device administers air into the nose safely and consistently. The device is simple and non-invasive. While swallowing, the air moves up the E.T. and cleans and ventilates the ME. The EarPopper alleviates negative pressure inside the ear, allowing the accumulated fluid to dry. According to the manufacturer, the EarPopper is the safest, simplest solution for ME inflammations. These claims are based on the results of a study diagnosing children with hearing loss due to MEE [5]. In the current study, we aimed to answer the question: what is the contribution of the EarPopper device to hearing improvement in children with MEE? We wished to evaluate the immediate change in pressure after using the device, as well as the pressure change over time (seconds to hours). In addition, we hoped to learn whether employing tympanometry immediately after the use of the device contributed to predicting success at the end of the treatment.

## 2. Materials and Methods

### 2.1. Participants

Participants in this study were children three years and older who had been diagnosed with MEE of at least three months duration with no other pathological finding per otoscopy by a senior Otologist (children with retraction or atelectasis or with glue ear were not included in the study in an attempt to assess a uniform patient population) and confirmed by tympanometry (At the time of the study, the manufacturer’s recommendation was for use in children over 1 year old). A preliminary examination (audiologic and otoscopic) of all participants (N = 91) prior to treatment (determining the presence of hearing impairment) was used as a baseline for the study. Three participants were excluded from the study because they showed spontaneous recovery during the initial examination before receiving the device, leaving 88 participants in the final study group. This group consisted of 37 males and 51 females, with a mean age of 5.4 years ± 3.5 years (median = 5). The control group consisted of 56 children with MEE who were followed without medical or surgical intervention. The control group included 30 males and 26 females, with a mean age of 5.7 years ± 1.95 years (median = 5).

### 2.2. Instrument

The self-politzerization device used in this study is the EarPopper device, EP3000 Pro (Summit Medical, St. Paul, MN, USA). There are two airflow settings (level I and level II), which represent the lower and higher airflow levels, respectively (a picture of the scheme of the device is in reference [5]). For this study, we used level I. The instruction to the participants was to take a sip of water and keep it in your mouth. Close one nostril with your finger, insert the device into the other nostril, and turn the device on. Then, swallow the water in your mouth. This study was conducted on young children, so parents performed the procedure to ensure that the device use was effective. 

The hearing measurements were tested by a GSI 61 Audiometer, Eden Prairie, MN, USA. ME pressure was measured by GSI TympStar, Eden Prairie, MN, USA.

### 2.3. Procedure

Part 1 was the six-week follow-up.

We compared the study group’s participants’ auditory exams before and after treatment to the control group’s participants’ auditory exams.

The assessments performed included an audiogram composed of a speech reception threshold (SRT) test, SRT by air conduction, air-conduction threshold and bone-conduction threshold at 250–4000 Hz, and tympanometry. The auditory exams were adjusted for age. In the young children, SRTs were tested with pictures and pure tones by play-conditioning audiometry. Older children were tested as adults. During the session, before the device was used, parents had received instructions on how to properly use the device. For the following six weeks, the study group used the device twice a day, twice in each nostril. The instruction included using the device for the last time the night before the follow-up auditory exams. The parents were instructed not to use the device during an active upper airway infection.

Part 2 was tympanometry before and immediately after using the EarPopper.

In order to evaluate the immediate change in pressure in the ME following the EarPopper procedure, tympanometry was performed on 21 participants, for a total of 42 ears, before and after performing the EarPopper procedure. The results were compared to the tympanometry results after 6 weeks of EarPopper use.

Part 3 was the length of the effect and an observational note.

One patient underwent tympanometry before, immediately after, 10 min later, and 90 min after using the EarPopper device. 

### 2.4. Statistical Analysis 

The mean and the standard deviation of the auditory exams and tympanometry results were analyzed before and after using the EarPopper device in the study group and compared with the control group. A three-way ANOVA test was used, and *p* ≤ 0.01 was considered significant. The Pearson correlation test was used to analyze the pressure measurement before and immediately after the first use of the device and was compared to the result after six weeks.

A three-way ANOVA test was used for the comparison between the right/left ear in the study group and the control group. All statistical analyzes were conducted using SPSS 23 software.

## 3. Results

### 3.1. Follow Up Six Weeks after Using the Device Twice a Day

#### 3.1.1. Difficulty in Using the Device over Time

Of the 88 participants’ parents, 16 (approx. 18%) were unable to use the device properly (complaints such as crying, pain, and “ear swelling”). The participants’ ages also had some effect on the ability to use the device, as described later. These complaints were likely part of the parents’ difficulty in the continued use of the device over an extended period of time. Five participants (approx. 5%) managed to use the device but did not adhere to using it throughout the six weeks required for follow-up. Another six participants (approx. 7%) did adhere to using the device and reported hearing improvement but did not return for the follow-up at the end of the treatment. One participant (approx. 1%) developed recurrent ear inflammations and could not use the device. Of the 88 participants who started to use the device, 60 completed the study (68%) in the study group. 

#### 3.1.2. Participants’ Age

We attempted to use it in a small number of children of three to four years of age, and out of four children, only one was able to comply with the use of the device. Of 21 children four to five years of age, 13 cooperated with using the device; at the age of five years and higher, age was no longer a factor.

The tympanometry results showed that a comparison of the pressure values in the ME before and after using the device presented a significant difference in the study group (*p* < 0.001). Type B was measured as −400 daPa, which allowed us to calculate the mean values for every mode of measurement. In the right ear, the mean value before use was −347 daPa (SD = 100), and the mean value after use was −173 daPa (SD = 159). In the left ear, the mean value before use was −340 daPa (SD = 134), and the mean value after use was −185 daPa (SD = 148), a change of 174 daPa and 155 daPa, respectively.

In the control group, a comparison of the pressure values at the beginning of the study and after six weeks did not show any significant difference between the two measurements (*p* = 0.11). In the right ear, the mean value at the beginning of the study was −336 daPa, and the mean value after six weeks was −315 daPa. In the left ear, the value at the beginning of the study was −355 daPa, and the mean value after six weeks was −322 daPa, a change of 21 daPa and 33 daPa, respectively. 

Another way to present the tympanometry results is to show how many ears changed their tympanometry type after using the device. 

**Figure 1:**
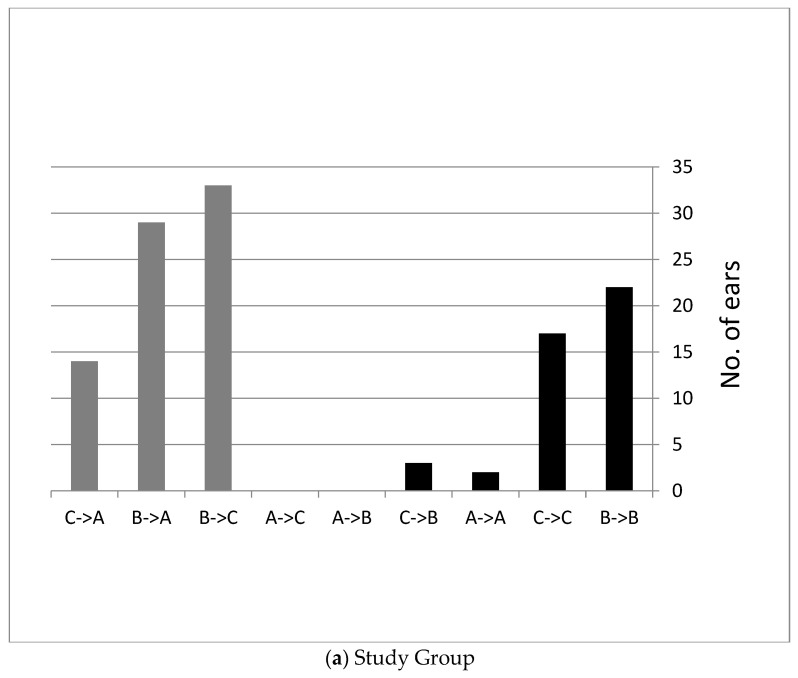

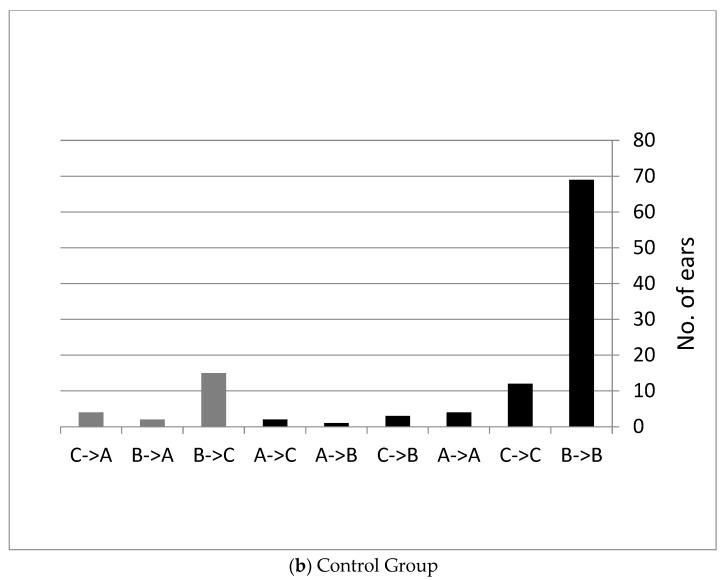
Distribution of tympanometry type in the study (**a**) and the control (**b**) groups after six weeks of EarPopper use. (Type A ≤ −100 daPa, −100 daPa >Type C > −400 daPa, Type B = −400 daPa).

Figure 1a shows the distribution of tympanometry type in the study group. In the study group, black ears showed worsening or no change during the study. In grey ears, the pressure improved after the use of the device (approximately 63% of the ears). 

Figure 1b shows the distribution of tympanometry type in the control group. In the control group, black ears showed worsening or no change during the study (approximately 82% of the ears showed worsening or remained unchanged during the study). In grey ears, the pressure improved after the use of the device.

##### Hearing Measurements

For the study group, the mean values of hearing measurement of air conduction were reviewed before and after using the device for six weeks. The average improvement in the hearing threshold ranged from 9.1 dB (2000 Hz—left ear) to 14 dB (500 Hz—right ear). PTA (500, 1000, and 2000 Hz) changed from 26 dB in both ears (mild hearing loss) to 13.1 dB in the right ear and 14.2 dB in the left ear (normal hearing). 

For the control group, the mean values of hearing measurement of air conduction were reviewed initially and after six weeks. The average improvement in the hearing threshold ranged from 1.1 dB (1000 Hz—left ear) to −0.9 dB (500 Hz—right ear). PTA (500, 1000, and 2000 Hz) has not changed since before and after using the device, with −25 dB on both ears (mild hearing loss). 

The results are presented in Table 1 and Table 2. Three participants in the study group and four in the control group were too young for full audiometry, so only SRT was presented for those patients.

To test the effects of group (study/control), time (before/after six weeks), and side (right/left), a three-way ANOVA was performed. The analysis revealed a main effect at all hearing thresholds, including the SRT threshold (air conduction), *F* (1, 112) = 10.09, *p* = 0.002, *η*^2^ = 0.09 at 250 Hz, *F* (1, 112) = 11.83, *p* = 0.001, *η*^2^ = 0.10 at 500 Hz, *F* (1, 112) = 15.82, *p* < 0.001, *η*^2^ = 0.13 at 1000 Hz, *F* (1, 112) = 9.95, *p* = 0.002, *η*^2^ = 0.09 at 2000 Hz, *F* (1, 112) = 13.05, *p* < 0.001, *η*^2^ = 0.11 at 4000 Hz, *F* (1, 112) = 21.17, *p* < 0.001, *η*^2^ = 0.16 in the SRT threshold, and a main effect in ME pressure *F* (1, 112) = 20.96, *p* < 0.001, *η*^2^ = 0.16. In the study group, the threshold was better than in the control group after six weeks. A significant main effect of **time** (or difference between initial and after six weeks) emerged at all hearing thresholds, including the SRT threshold (air conduction), *F* (1, 112) = 45.91, *p* < 0.001, *η*^2^ = 0.30 at 250 Hz, *F* (1, 112) = 45.22, *p* < 0.001, *η*^2^ = 0.30 at 500 Hz, *F* (1, 112) = 46.21, *p* < 0.001, *η*^2^ = 0.30 at 1000 Hz, *F* (1, 112) = 36.48, *p* < 0.001, *η*^2^ = 0.25 at 2000 Hz, *F* (1, 112) = 23.44, *p* < 0.001, *η*^2^ = 0.22 at 4000 Hz, *F* (1, 112) = 54.07, *p* < 0.001, *η*^2^ = 0.33 in the SRT threshold, and a main effect in ME pressure *F* (1, 112) = 64.53, *p* < 0.001, *η*^2^ = 0.36.

The analysis revealed a significant interaction of group × time at all hearing thresholds, including the SRT threshold (air conduction), *F* (1, 112) = 52.96, *p* < 0.001, *η*^2^ = 0.33 at 250 Hz, *F* (1, 112) = 52.96, *p* < 0.001, *η*^2^ = 0.33 at 500 Hz, *F* (1, 112) = 36.91, *p* < 0.001, *η*^2^ = 0.26 at 1000 Hz, *F* (1, 112) = 23.92, *p* < 0.001, *η*^2^ = 0.18 at 2000 Hz, *F* (1, 112) = 32.99, *p* < 0.001, *η*^2^ = 0.24 at 4000 Hz. *F* (1, 112) = 40.06, *p* < 0.001, *η*^2^ = 0.26 in the SRT threshold. In addition, a significant interaction of ME pressure measured by tympanometry emerged, *F* (1, 112) = 32.68, *p* < 0.001, *η*^2^ = 0.22. A post hoc Bonferroni analysis of interaction revealed that, in the control group, there was no difference between the measurement initially and after six weeks (*p* = 0.73 at 250 Hz, *p*= 0.76 at 500 Hz, *p* = 0.62 at 1000 Hz, *p* = 0.43 at 2000 Hz, *p* = 0.83 at 4000 Hz, *p* = 0.48 in the SRT threshold, and *p* = 0.11 in ME pressure), whereas in the study group there was a significant difference between before and after using the device. In other words, the hearing threshold was better at all frequencies, and the ME pressure was less negative after using the device (p< 0.001 in all frequencies and pressure).

There was no significant difference between right and left in all hearing thresholds, including SRT threshold (air conduction), *F* (1, 112) = 2.09, *p* = 0.15 at 250 Hz, *F* (1, 112) = 0.59, *p* = 0.45 at 500 Hz, *F* (1, 112) = 0.38, *p* = 0.54 at 1000 Hz, *F* (1, 112) = 0.09, *p* = 0.77 at 2000 Hz, *F* (1, 112) = 0.07, *p* = 0.79 at 4000 Hz, *F* (1, 112) = 0 in the SRT threshold, and ME pressure by tympanometry, *F* (1, 112) = 0.1, *p* = 0.69.

The interaction of group × side was not significant for all hearing thresholds, including the SRT threshold (air conduction), *F* (1, 112) = 0.05, *p* = 0.83 at 250 Hz, *F* (1, 112) = 0 at 500 Hz, *F* (1, 112) = 0 in 1000 Hz, *F* (1, 112) = 0.06, *p* = 0.81 in 2000 Hz, *F* (1, 112) = 0.01, *p* = 0.91 at 4000 Hz in SRT threshold, *F* (1, 112) = 0.07, *p* = 0.77, and there was no interaction with the ME pressure measured by tympanometry, *F* (1, 112) = 0.46, *p* = 0.50. The interaction of time × side was not significant for all hearing thresholds, including the SRT threshold (air conduction), *F* (1, 112) = 0.20, *p* = 0.65 at 250 Hz, *F* (1, 112) = 0 at 500 Hz, *F* (1, 112) = 0 at 1000 Hz, *F* (1, 112) = 0.13, *p* = 0.72 at 2000 Hz, *F* (1, 112) = 0.94, *p* = 0.34 at 4000 Hz, in the SRT threshold, *F* (1, 112) = 0.26, *p* = 0.61. Furthermore, there was no interaction with ME pressure measured by tympanometry, *F* (1, 112) = 0.49, *p* = 0.48. 

### 3.2. Tympanometry before and Immediately after Using EarPopper (Tympanometry during the First Use of the Device)

**Figure 2:**
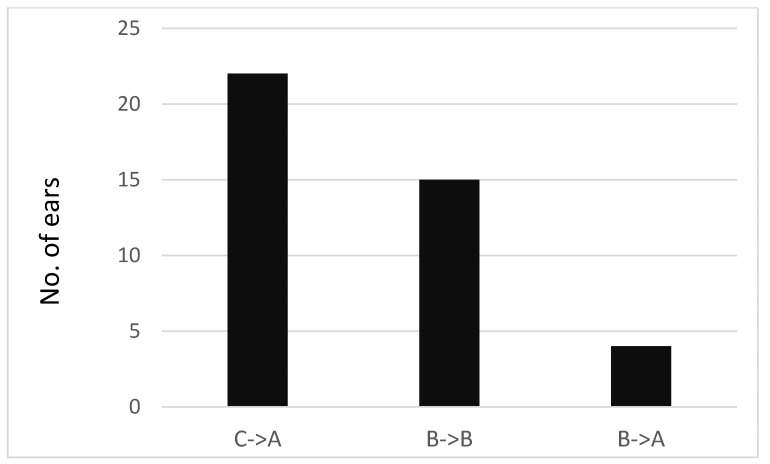
Tympanometry results before and immediately after using the device. (Type A ≤ −100 daPa, −100 daPa >Type C > −400 daPa, Type B = −400 daPa).

Tympanometry was performed on 21 study participants for a total of 42 ears before and after performing the EarPopper procedure. One participant had normal pressure in the right ME (Type A). The results are presented for 41 ears. The mean pressure value before use was −310 daPa in the right ear and −296 daPa in the left ear, and the mean value immediately afterward was −76 daPa and −138 daPa, respectively. The difference between before the EarPopper procedure and immediately after was found to be significant {right: *t* (19) = 5.59, *p* < 0.001, left: *t* (20) =4.48, *p* < 0.001} Looking at a change in tympanometry type, in participants with negative pressure (Type C), an immediate improvement toward normal pressure in the ME (Type A) was observed (22 of 22 ears). In Type B, no immediate change in pressure was observed in most cases (15 out of 19 ears). Only four ears changed from Type B to Type A (Figure 2).

### 3.3. Length of Effect

Tympanometry was conducted before using the device and up to 1 ½ h afterward. A follow-up over time was performed on one participant using tympanometry. The follow-up results (two ears) showed that the positive pressure introduced into the ear by the device was not maintained over time. After 1 ½ h, both ears tested for negative pressure. The results are presented in Table 3.

Pearson correlation analyses were performed between the pressure measurement results initially and after six weeks. A medium positive significant correlation emerged for the right ear, *r* = 0.57, *p* = 0.01, and a high positive correlation emerged for the left ear, *r* = 0.79, *p* < 0.001. 

Correlations between an immediate improvement in pressure and the pressure measurement results after six weeks were not significant (Pearson correlation, right ear: *r* = 0.23, *p* = 0.34, left ear: *r* = 0.28, *p* = 0.23). 

## 4. Discussion

The EarPopper device administers air into the nose safely, consistently, and in a controlled manner. These claims are backed by a small number of studies [17,18,19,20]. 

In this independent work, we sought to evaluate the short and intermediate effects of the EarPopper device on ME status and hearing in children with MEE. In part 1 of the study, for the study group, a significant change was found in pressure values based on tympanometry and improvement in the hearing thresholds before and after using the device. For the control group, no significant change was found in pressure values nor improvement in the hearing thresholds. We, therefore, assume that the change in the study group is due to the use of the device and that continuous use over six weeks had beneficial effects on MEE. In our study, the improvement in the hearing threshold average ranged from 9.1 dB (2000 Hz—left ear) to 14 dB (500 Hz—right ear). The measurements in the study carried out by Banigo et al., (2016) [20] compared to our measurements (8.2 dB 500 Hz for both ears to 14.8 dB 4000 Hz for both ears. The manufacturers [5] found that their average improvement was greater than ours in the hearing threshold range from 14.8 dB (500 Hz—right ear) to 18.3 dB (500 Hz—left ear). A comparison of the pressure values in the ME before and after using the device also showed a significant difference in the study group: a change of 174 daPa in the right ear and 155 daPa in the left ear. These results were in accordance with the manufacturer’s study: a change of 112 daPa in the right ear and 156 daPa in the left ear [5]. 

In part 2 of this study, we examined the immediate effect of EarPopper use. With initial use, all ears with Type C tympanometry turned into Type A after a single use. Ears with Type B tympanometry seemed to be largely unaffected, with only four of nineteen ears becoming Type A. Although tympanometry in most Type B ears did not change after a single use, the four that did improve from Type B to Type A indicated that the device’s use can have an immediate effect. These initial results (in part 2 of our study) would appear to suggest that an initial trial of the device would predict outcomes or detect who might benefit from the use of the device. However, as there was no correlation between those who appeared to respond to the initial treatment and those who showed improvement in part 1, we concluded that the long-term effect of the device depended on a cumulative process that could not be predicted after a single use. 

In part 3, we examined the change in ME pressure after the initial use of EarPopper in one participant with Type B and C tympanometry. The pressure measurement over time in this participant, before introducing the air pressure with the device and up to 90 min afterward showed that pressure decreased back to negative values, and this process took place within minutes. E.T. ventilation is not the exclusive cause of ME aeration. Pressure balance is kept within normal ranges with the combined effect of E.T., mucosal gas diffusion, and the buffering effect of mastoid cells [21]. Sade et al. (1995) [22] found that, when atelectatic ears are politzerized (hyperinflated) with different gases, these gases disappear at a speed that corresponds to the diffusion coefficient of those gases. This suggests that there is a diffusional process from the ME into the blood and in the opposite direction that mainly determines the ME pressure. It is possible that the reason that part of the patients’ hearing thresholds or ME pressure in our study did not improve, although gas was inserted into the ME by the EarPopper, was that it was quickly absorbed. Since this effect was measured in only one patient, we cannot draw a valid conclusion about the mechanism involved.

It is important to note that the initial tympanometry results in part 2 of the study did not correlate with the results in part 1. Thus, the initial change in tympanometry type while using the EarPopper did not predict the success in the treatment of MEE in the long run. Therefore, we recommend that an initial assessment of the efficacy of the device’s use should take place after a few weeks of practice. Further study should be done to determine a precise time for initial assessment that might be a predictor for long-term response to the treatment. Another important issue is compliance with the use of the EarPopper device. In our study, only one participant under the age of four was able to comply with the regular use of the EarPopper. At the ages of five years and above, compliance was no longer a limiting factor. Our results suggest that the EarPopper is not an alternative to traditional treatment for children under four years. This recommendation was amended in recent years to 4+ based on the results of studies similar to the current study. This is an important point, as the majority of MEE cases present under the age of three years. However, in children above four years old, the device appears to be effective, as described in a previous study [23]. There was a significant improvement in hearing thresholds, including the SRT threshold and the ME status in the study group, compared with controls. This finding supports the impression that the EarPopper can be offered as a treatment option for children with MEE and conductive hearing loss. The strength of this recommendation is affected, of course, by the limitations of our study. It is important to note that, although there was a significant improvement in hearing thresholds and ME status in the study group, neither appear to be completely normal. ME pressure is still outside the normal range, and the hearing thresholds suggest a mild conductive loss, at least at some frequencies. We cannot speculate on the length of effect or whether longer use provides a higher level of improvement. Our study did not head-to-head compare the use of the device with the insertion of tympanostomy tubes. This is probably the most important study needed, as it would establish the value of the EarPopper device as a valid alternative to surgery.

Other points that need to be addressed in further studies are the length of use and the timing of follow-up. We cannot confirm the durability of the treatment or how long the improvement lasts, as we did not follow up on the ME status after six weeks. A recommendation for the treatment period would need to be based on a study with a larger study group and a comparison of several intervals of device use. The study should also include a follow-up after the discontinuation of the device in order to ascertain the length of effect. Follow-up periods and timing of the first follow-up after beginning use of the device are important issues raised by the findings in our study. As the initial tympanometry results in part 2 of the study did not correlate with the results in part 1 and cannot be used as a predictor for long-term response, it is suggested that the initial evaluation of response not be done after a single use of the device. A simple way to establish the follow-up time frame is to carry out a study in which tympanometry would be performed every week during the device’s use. This would enable the assessment of the minimal time period, where early tympanometry predicts long-term response. Children who fail to show improvement at the minimal follow-up time should discontinue the treatment and be referred for surgery. 

## Figures and Tables

**Table 1:** Auditory thresholds before and after six weeks.

Study Group (N = 57)	Right Ear	Frequency	250 Hz	500 Hz	1000 Hz	2000 Hz	4000 Hz
		MTB * (SD)	32.9 (11.4)	31.4 (12.2)	27.0 (11.9)	18.5 (11.8)	24.2 (13.3)
		MTA ** (SD)	19.2 (10.8)	17.4 (9.0)	13.5 (8.0)	8.4 (7.6)	11.5 (8.2)
	Left ear	Frequency	250 Hz	500 Hz	1000 Hz	2000 Hz	4000 Hz
		MTB * (SD)	34.0 (11.9)	31.8 (11.2)	27.1 (11.4)	18.5 (12.0)	23.6 (13.8)
		MTA ** (SD)	20.8 (8.6)	18.6 (7.8)	14.7 (9.0)	9.4 (7.7)	12.4 (8.9)
Control Group (N = 52)	Right ear	Frequency	250 Hz	500 Hz	1000 Hz	2000 Hz	4000 Hz
		MTB * (SD)	30.0 (9.1)	28.7 (9.7)	26.1 (12.0)	18.9 (10.6)	23.6 (12.3)
		MTA ** (SD)	30.2 (12.2)	29.6 (13.6)	25.8 (13.7)	18.0 (12.0)	23.4 (14.1)
	Left ear	Frequency	250 Hz	500 Hz	1000 Hz	2000 Hz	4000 Hz
		MTB * (SD)	31.5 (10.0)	30.0 (11.6)	27.2 (11.9)	19.0 (11.7)	23.5 (11.8)
		MTA ** (SD)	32.3 (11.8)	29.9 (10.4)	26.1 (11.3)	18.0 (11.7)	24.3 (12.6)

* MTB—mean threshold, dB, before using the device. ** MTA—mean threshold, dB, after using the device.

**Table 2:** SRT before and after six weeks.

Study Group (N = 60)	Right Ear	SRT	
		MTB * (SD)	22.5
			(11.3)
		MTA ** (SD)	9.7
			(6.1)
	Left ear	SRT	
		MTB * (SD)	22.2
			(9.5)
		MTA ** (SD)	10.7
			(7.0)
Control Group (N = 56)	Right ear	SRT	
		MTB * (SD)	22.7
			(11.2)
		MTA ** (SD)	21.9
			(11.5)
	Left ear	SRT	
		MTB * (SD)	22.6
			(10.9)
		MTA ** (SD)	21.6 (10.1)

* MTB—mean threshold, dB, before using the device. ** MTA—mean threshold, dB, after using the device.

**Table 3:** Follow-up over time.

	Right Ear (daPa Pressure)	Left Ear (daPa Pressure)
Before use	−400	−124
Immediately after use	+44	+124
10 min after use	−116	+44
1.5 h after use	−268	−56

## Data Availability

The data presented in this study are available on request from the corresponding author. The data are not publicly available due to privacy or ethical.
